# A mixture model to detect edges in sparse co-expression graphs with an application for comparing breast cancer subtypes

**DOI:** 10.1371/journal.pone.0246945

**Published:** 2021-02-11

**Authors:** Haim Bar, Seojin Bang

**Affiliations:** 1 Department of Statistics, University of Connecticut, Storrs, CT, United States of America; 2 Computational Biology Department, Carnegie Mellon University, Pittsburgh, PA, United States of America; University Campus Bio-Medico of Rome, ITALY

## Abstract

We develop a method to recover a gene network’s structure from co-expression data, measured in terms of normalized Pearson’s correlation coefficients between gene pairs. We treat these co-expression measurements as weights in the complete graph in which nodes correspond to genes. To decide which edges exist in the gene network, we fit a three-component mixture model such that the observed weights of ‘null edges’ follow a normal distribution with mean 0, and the non-null edges follow a mixture of two lognormal distributions, one for positively- and one for negatively-correlated pairs. We show that this so-called *L*_2_ *N* mixture model outperforms other methods in terms of power to detect edges, and it allows to control the false discovery rate. Importantly, our method makes no assumptions about the true network structure. We demonstrate our method, which is implemented in an R package called *edgefinder*, using a large dataset consisting of expression values of 12,750 genes obtained from 1,616 women. We infer the gene network structure by cancer subtype, and find insightful subtype characteristics. For example, we find thirteen pathways which are enriched in each of the cancer groups but not in the Normal group, with two of the pathways associated with autoimmune diseases and two other with graft rejection. We also find specific characteristics of different breast cancer subtypes. For example, the Luminal A network includes a single, highly connected cluster of genes, which is enriched in the human diseases category, and in the Her2 subtype network we find a distinct, and highly interconnected cluster which is uniquely enriched in drug metabolism pathways.

## Introduction

Broadly speaking, statistical analysis of ‘omics’ data consists in studying the relative abundance of biological ‘building blocks’, such as genes, proteins, and metabolites. The goal of many studies involving high-throughput data is to identify differential building blocks—those whose abundance levels vary according to the value of some other factor. These factors include, for example, environmental conditions, disease state, gender, or age. To simplify the discussion, we will use genomics terminology, where the building blocks are genes and the abundance is their expression levels. Many biological studies use statistical methods which focus on individual genes and rely on the unrealistic, but mathematically convenient assumption that the expression levels are independent across genes. However, other methods drop this assumption and acknowledge that multiple genes are likely to work as a group associated with the same biological process, thus providing not only more complex functionalities, but also robustness to detrimental mutations. A common assumption is that related genes share a regulatory process, and therefore, their expression levels are expected to be highly correlated.

Our motivating example is a large dataset consisting of expression values of 12,750 genes obtained from 1,616 women. We are particularly interested in inferring the gene network structure by cancer subtype, where the possible categories are Basal, Her2 Positive, Luminal A, and Luminal B. The dataset also contains a sample of healthy women. Like most ‘omics’ datasets, the one being investigated here contains a large number of genes, and therefore, a very large number of possible edges (over 80 million, in the case presented in this paper), which makes discovering the network structure quite challenging. In biological systems gene networks are expected to be sparse [1], but how can we decide which of the millions of gene pairs are indeed highly correlated? This entails statistical challenges as well as computational ones. Namely, which mathematical model should be used in order to unveil as many strongly correlated gene pairs as possible (‘true-positive edges’), while keeping the number of ‘false-positive edges’ small; and how can we perform the necessary computations efficiently? Our objective in this paper is to present a novel approach, and to demonstrate interesting insights obtained from our gene networks analysis when applied to each breast cancer subtype. The approach presented here is implemented in an R package called edgefinder.

In an undirected graph representation of a gene network, genes correspond to nodes and each edge is assigned a weight based on the strength of the association between the corresponding pair of genes. Therefore, the key to any gene network analysis method is to quantify the notion of ‘strength of the association’, or *co-expression* levels between pairs of genes. Then, in order to achieve a sparse network, we have to define a threshold, below which the weight of the edge is assumed to be 0. Ideally, such a threshold should be accurate in the sense that any edge that has been removed (i.e., weight = 0) corresponds to a pair of truly unrelated genes, and any edge that has been retained corresponds to a truly correlated pair.

In this paper we measure co-expression in terms of Pearson’s correlation coefficient, but other quantifications may be considered, including mutual information, Spearman’s rank correlation coefficient, or Euclidean distance. Our approach is described in more detail in the next section, but briefly, to address the statistical and computational challenges our approach is the following: in order to obtain the weights, *w*_*ij*_, we apply Fisher’s Z transformation to each pair’s sample correlation coefficient, *r*_*ij*_. For uncorrelated pairs the *asymptotic* distribution of *w*_*ij*_ is normal, with mean zero and variance 1/(*N* − 3). This motivates fitting a mixture model to {*w*_*ij*_} in which the majority of pairs belong to a normally distributed ‘null component’, and a small percentage of the weights belong to one of two ‘non-null components’, which follow log-normal distributions (one for positive and one for negative correlations). This so-called *L*_2_ *N* model was first presented in [2], in the context of identifying differentially expressed (or dispersed) genes. We use the *L*_2_ *N* model to recover a sparse gene network for the following reasons. First, this mixture model leads to shrinkage estimation and to borrowing strength across all pairs, which increases the power to detect co-expressed pairs. Second, the specific form of the mixture model allows us to establish an appropriate threshold for the weights such that we can control the error rate. Third, the mixture model lends itself to a computationally-efficient estimation of the parameters via the EM algorithm [3].

**Literature review**: Using gene co-expression patterns, a number of authors defined ‘modules’ as sets of genes that have similar expression patterns; they then focus on a small number of intramodular ‘eigengenes’ or ‘hub genes’ instead of on thousands of genes [4–8]. Among them, the weighted gene co-expression network analysis (WGCNA, [5]) is a widely used approach. Among hub genes, the aforementioned independence assumption is more reasonable because genes belonging to different modules are expected to be much less correlated than genes within the same module. Thus, one can try to find differentially expressed hub genes with respect to a trait or treatment. However, methods assuming a specific structure and focusing on modules and their eigengenes or hub genes, are only suitable for certain networks where nodes within a module are highly connected but connections across modules are relatively rare. Biological networks such as protein-protein and gene-gene interaction networks may have other network features where modules cannot be clearly partitioned. Such is the case, for example, with scale-free networks [9–11], a scale-free regime followed by a sharp cutoff [12–14], and other networks with curved degree distributions [15–18].

Instead of identifying hub genes and comparing their differential expression levels between two traits or treatments, there have been other approaches that compare high dimensional covariance matrices between the two groups and identify risk genes based on the correlation structures [19–23]. Obtaining accurate estimates of covariance (or precision) matrix is important for gene network analyses, but it becomes challenging when the number of genes is larger than the sample size (as is often the case). The problem of estimating large, sparse gene networks has been thoroughly studied in modern multivariate analysis. Researchers have suggested various approaches using regularization techniques, and one of most commonly used approaches is the penalized maximum likelihood. For example, [24] proposed to estimate a precision matrix by imposing an *l*_1_ penalty on a Gaussian log-likelihood to increase its sparsity. It uses a simple algorithm to estimate a sparse precision matrix, by fitting a regression model to each variable with all other variables as predictors, and apply the lasso to obtain sparsity. [25] suggested a simple but faster algorithm called graphical lasso, which also estimates a sparse precision matrix using coordinate descent procedure for lasso. [26–29] also proposed algorithms to solve the *l*_1_ penalized Gaussian log-likelihood to estimate a large, sparse precision matrix. These methods have been extensively used to estimate sparse gene network. Recently, [22] suggested a method for large-scale testing of correlations under certain regularity conditions. [23] suggested a sparse leading eigenvalue driven test that compares two high-dimensional covariance matrices obtained from schizophrenia and normal groups and identified novel schizophrenia risk genes.

Genes that are found to be highly associated with a trait or treatment are further analyzed by using knowledge-based pathway analysis tools such as Gene Set Enrichment Analysis (GSEA, [30, 31]). Pathway analysis differs from co-expression analysis in that it uses *pre-defined* gene sets (e.g., from the Gene Ontology (GO, [32]) or the Kyoto Encyclopedia of Genes and Genomes (KEGG, [33]). Such analyses help determine which pathways are over- or under-represented in the identified modules [34].

For a more detailed review of related methods, we refer the reader to [8].

## Statistical model and estimation

### A mixture model for edge indicators in a gene network

A gene network can be represented by a weighted, undirected graph in which each node corresponds to a gene and each edge corresponds to a pair of genes that are ‘co-expressed’, meaning that their expression levels are highly correlated. The weights represent the strength of the connection between two genes, namely, their tendency to be co-expressed. Given normalized expression data of *G* genes, our objective is to discover the network structure, namely, which of the *K* = *G*(*G* − 1)/2 pairs are co-expressed. Our general strategy is to associate with every putative edge in the complete graph with *G* nodes, a latent indicator variable whose value (0 or 1) is determined by a statistical model.

To start, we define the edge weights in terms of pairwise correlation coefficients. Let **x**_*g*_ be a vector of normalized expression levels for gene *g* ∈ {1, …, *G*} obtained from *N* samples (*N* > 3). Suppose that the true correlation coefficient between genes *m* and *n* is *ρ*_*mn*_, and let *r*_*mn*_ = *corr*(**x**_*m*_, **x**_*n*_) be the observed correlation coefficient. Using Fisher’s Z transformation, we obtain the estimated weight *w*_*mn*_ = *arctanh*(*r*_*mn*_), which is known to be approximately normally distributed, with mean *arctanh*(*ρ*_*mn*_) and variance $\frac{1}{N-3}$. Let *E* = {*e*_*mn*_} be the set of true edges in the network. We assume that *G* is large and that most pairs are not co-expressed, so the network is sparse: |*E*| ≪ *K*. This assumption, along with asymptotic normality of *w*_*mn*_, motivate our model choice. Specifically, we assume that the weights follow the so-called *L*_2_ *N* mixture distribution in [2]. *L*_2_ *N* is a three-component mixture model in which the ‘null’ component follows a normal distribution with mean 0, representing the majority of pairs that have approximately zero correlation, and the tails (the ‘non-null’ components, for pairs with strong positive/negative correlations) follow log-normal distributions:

$$\begin{array}{ccc} w_{mn}|e_{mn}\notin E & ∼ & N(0,\sigma^{2}) \end{array}$$ (1)

$$\begin{array}{ccc} w_{mn}|\left[w_{mn}>0,\;e_{mn}\in E\right]\; & ∼ & LogNormal(\theta_{1},\kappa_{1}^{2})\;, \end{array}$$ (2)

$$\begin{array}{ccc} -w_{mn}|\left[w_{mn}<0,\;e_{mn}\in E\right]\; & ∼ & LogNormal(\theta_{2},\kappa_{2}^{2})\;. \end{array}$$ (3)

**Fig 1:**
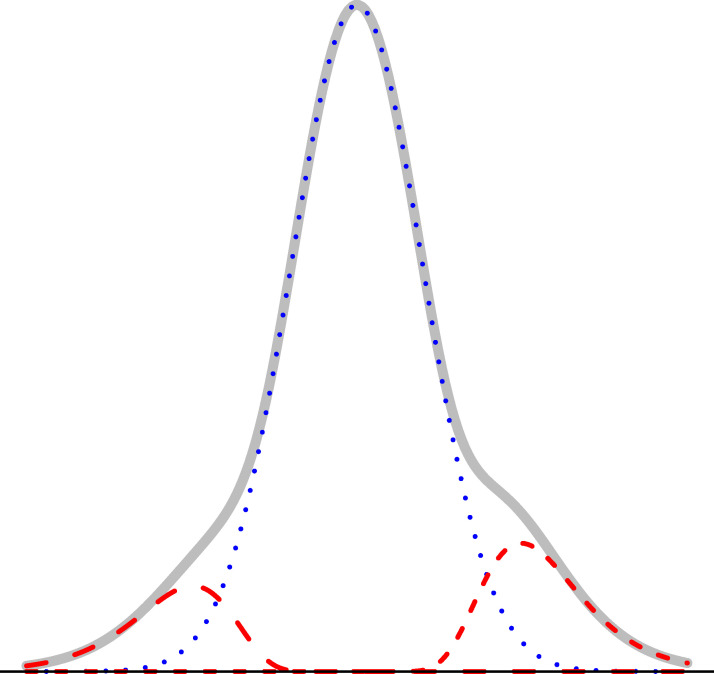
The *L*_2_ *N* mixture model with probability of the null component (blue dotted curve), *Pr*(*mn* ∈ *C*_0_) = 0.8, where *mn* denotes the edge between nodes *m* and *n* in the graph.

Note that *σ*^2^ consists of two variance components, namely, $\sigma^{2}=\frac{1}{N-3}+\sigma_{0}^{2}$, where $\frac{1}{N-3}$ is the variance component due to the asymptotic distribution of *arctanh*(*r*_*mn*_), whereas $\sigma_{0}^{2}$ is due to the random effect model, which allows us to account for extra variability among uncorrelated pairs. A graphical representation of the *L*_2_ *N* model is shown in Fig 1.

If we denote the null mixture component in the *L*_2_ *N* model by *C*_0_, the two non-null components by *C*_1_ and *C*_2_, the corresponding probability density functions by *f*_*j*_, and the mixture probabilities by *p*_*j*_, for *j* = 0, 1, 2, such that *p*_0_ + *p*_1_ + *p*_2_ = 1, then we classify a putative edge between nodes *m* and *n* in the complete graph into one of the three mixture components based on the posterior probabilities,

$$\begin{array}{c} Pr\left(e_{mn}\in C_{j}|w_{mn}\right)=\frac{p_{j}f_{j}\left(w_{mn}\right)}{p_{0}f_{0}\left(w_{mn}\right)+p_{1}f_{1}\left(w_{mn}\right)+p_{2}f_{2}\left(w_{mn}\right)},\;j=0,1,2. \end{array}$$ (4)

Note that when *G* is large, the total number of possible edges for which we have to evaluate Eq (4) is quite large. In the Implementation Notes section below, we propose a sampling approach which ensures computational efficiency. Let **b**_*mn*_ = (*b*_0*mn*_, *b*_1*mn*_, *b*_2*mn*_) be an indicator vector, so that *b*_*jmn*_ = 1 for the component *j* with the highest probability, *Pr*(*e*_*mn*_ ∈ *C*_*j*_|*w*_*mn*_), for the pair *mn*, and 0 for the other two components. Using this notation, the *G* × *G* matrix **A** = [1 − *b*_0*mn*_] denotes the *adjacency matrix* between the *G* nodes in the graph. Our goal is to obtain an accurate estimate of **A**. To do that, we treat the indicators **b**_*mn*_ as missing data, and use the EM algorithm [3] to estimate the parameters of the mixture model. The hierarchical and parsimonious nature of the *L*_2_ *N* model leads to shrinkage estimation and borrowing power across all pairs of genes, as well as to computational efficiency. This is critical, since *K* is typically very large and can be much larger than the sample size, *N*. Details regarding the parameter estimation for the *L*_2_ *N* model can be found in [2]. The algorithm is implemented as an R-package called edgefinder, available from github (github.uconn.edu/hyb13001/edgefinder). For up to date documentation, see the file vignettes/edgefinder.pdf in the github repository. An earlier version of our work was posted on arXiv [35].

### Rationale for assuming sparsity in the correlation matrix

Other methods, some of which were mentioned in the introduction, assume sparsity in precision matrix. The use of precision matrix for estimating gene network is justified when the data is generated from a multivariate normal distribution. Under this assumption, elements of the precision matrix correspond to conditional independence restrictions between nodes in a gene network. When fitting a multivariate normal model, the likelihood function involves the precision matrix, hence assuming sparsity in this, rather than the covariance matrix, is convenient. Methods which assume a sparse precision matrix in a multivariate normal model work well when analyzing gene data obtained from large retrospective studies such as the TCGA data [36]. However, applying these approaches to datasets with a relatively small number of samples may not produce robust estimates.

Rather than estimating the precision matrix, we use the correlation matrix to determine which genes are co-expressed. Sparsity in the correlation matrix (rather than the precision matrix) is motivated mostly by biological arguments:

1. expression level of genes are associated with their functionality, and thus, are expected to be highly correlated for genes that share the same functional process; and
2. the number of genes associated with most biological functions is small, relative to the total number of genes.

There is also a strong mathematical argument for modeling sparsity in the covariance matrix, rather than the precision matrix. For each gene, its expression profile is an *N*-dimensional vector where *N* is the sample size, and for each pair of genes the correlation between their expression profiles represents the cosine of the angle between the two corresponding vectors. So, the correlation matrix offers an intuitive representation of the similarity between expression profiles. As [37] showed, as *N* increases, any two random vectors in the Euclidean *N*-dimensional space are approximately orthogonal with probability that approaches 1. Thus, the correlation between two random expression profiles is expected to be close to *cos*(*π*/2) = 0, which means that the correlation (and hence, the covariance) matrix is expected to be sparse.

### Implementation notes

There are a couple of challenges related to the parameter estimation via the EM algorithm which should be addressed. First, in order to obtain parameter estimates for the *L*_2_ *N* model from the complete-data log-likelihood, it was assumed that the normalized weights, *w*_*mn*_, are mutually independent. Conditionally, they are all asymptotically normally distributed with variance $\frac{1}{N-3}$, but for a fixed *m*, some *w*_*mn*_ may be correlated. Second, obtaining estimates based on all *K* pairwise correlations is time-consuming and requires storing a very large matrix in the computer’s memory, since the values of the indicator variables, ${\hat{b}}_{jmn}$, for each pair of genes must be updated in each iteration of the EM algorithm.

When the number of genes is large, as is the case with the breast cancer dataset which is the focus of this paper, we propose taking a random sample of *G*′ < *G* genes (e.g., *G*′ = 1000) and fitting the mixture model to this random subset. Using the smaller subset of genes allows us to assume that the *K*′ = *G*′(*G*′ − 1)/2 weights which correspond to pairs from the selected subset *are* approximately independent. It also greatly improves computational efficiency, since only *K*′ ≪ *K* posterior probabilities have to be computed in each iteration, and the resulting *L*_2_ *N* model parameters are highly accurate. With the estimates obtained from the random subset, it is then possible to compute the remaining *K* − *K*′ posterior probabilities (4) only once. This approach is feasible even on a computer with standard memory capacity.

In addition to computational efficiency and increasing power through shrinkage estimation, the mixture model allows us to estimate how many pairs of genes are correctly (or incorrectly) classified as co-expressed. Specifically, for a predetermined posterior probability ratio threshold, *T* > 1, we find *c*_1_ and *c*_2_ such that

$$\begin{array}{c} c_{1}=\text{arg}\underset{w\in (0,\infty )}{\text{min}}\frac{{\hat{p}}_{1}{\hat{f}}_{1}\left(w\right)}{{\hat{p}}_{0}{\hat{f}}_{0}\left(w\right)}>T\;\text{and}\;c_{2}=\text{arg}\max_{w\in (-\infty ,0)}\frac{{\hat{p}}_{2}{\hat{f}}_{2}\left(w\right)}{{\hat{p}}_{0}{\hat{f}}_{0}\left(w\right)}>T \end{array}$$

and set *b*_0*mn*_ = 1 if *w*_*mn*_ ∈ [*c*_2_, *c*_1_], and *b*_0*mn*_ = 0 otherwise. Alternatively, for any *α*, we can (numerically) find thresholds *c*_1_ > 0 and *c*_2_ < 0 such that

$$\begin{array}{ccc} Pr({\hat{b}}_{0mn}\neq 0\;|\;b_{0mn}=0) & \approx & {\hat{p}}_{0}\int_{-\infty }^{c_{2}}{\hat{f}}_{0}\left(w\right)dw+{\hat{p}}_{0}\int_{c_{1}}^{\infty }{\hat{f}}_{0}\left(w\right)dw\leq \alpha \;. \end{array}$$ (5)

That is, we control the estimated probability of a Type I error at a certain level, *α*. Similarly, we can control the false discovery rate [38]. Using the thresholds *c*_1_ and *c*_2_, we can estimate the probability of a Type II error:

$$\begin{array}{ccc} Pr({\hat{b}}_{0mn}=0\;|\;b_{0mn}\neq 0) & \approx & {\hat{p}}_{2}\int_{c_{2}}^{0}{\hat{f}}_{2}\left(w\right)dw+{\hat{p}}_{1}\int_{0}^{c_{1}}{\hat{f}}_{1}\left(w\right)dw. \end{array}$$ (6)

## Simulation study

### Data generated under the *L*_2_ *N* model

In the first simulation study, we assess the power and goodness of fit of our model using different configurations, with varying numbers of genes (G), samples (N), degrees of sparsity (*p*_1_ + *p*_2_), and graph structures. In this section, data are generated from the *L*_2_ *N* model, and we use different parameters for the log-normal components. We show representative results with *N* = 100 and *G* = 500 (thus, *K*, the maximum possible number of edges is 124,750.) Four network configurations are used in this section. They are described in terms of the shape of the *G* × *G* adjacency matrix, *A*, as follows:

- **complete**: *A* = *BlockDiag*(*J*_*S*_ − *I*_*S*_, 0_*G* − *S*_), where *S* = 100, *J* is a matrix of 1’s, *I* is an identity matrix, and 0 is a matrix of zeros. This graph contains one clique (complete subgraph) with 100 nodes, and nodes not in the clique are not connected to other nodes. |*E*| = 4, 950 (≈ 0.04*K*), *p*_1_ = 0.0396, *p*_2_ = 0.
- **ar** (autoregressive): *A* has a Toeplitz structure, with *A*_*ij*_ = 1/(1 + |*i* − *j*|) if both *i*, *j* ≤ *S*, where *S* = 100. |*E*| = 4, 950, *p*_1_ = 0.0396, *p*_2_ = 0.
- **two independent blocks**: *A* = *BlockDiag*(*J*_*S*_ − *I*_*S*_, *J*_*S*_ − *I*_*S*_, 0_*G* − 2*S*_), where *S* = 50. (I.e., two distinct cliques, each with 50 genes.) |*E*| = 2, 450, *p*_1_ = 0.0196, *p*_2_ = 0.
- **two negatively correlated blocks**: Similar to the previous configuration, but the two blocks are negatively correlated. |*E*| = 4, 950, *p*_1_ = 0.0196, *p*_2_ = 0.02.

For pairs *i*, *j* such that *A*_*ij*_ = 0, we generated *w*_*ij*_ independently from a standard normal distribution. In the *complete* and *two independent blocks* configurations, for *A*_*ij*_ = 1 we generated only positively correlated pairs (so *p*_2_ = 0), and in the *two negatively correlated blocks* configuration, the pairs were positively correlated within each block, but pairs across the two blocks were generated to be negatively correlated. For the *ar* structure, weights generated from the log-normal distribution appear in the off-diagonals of *A* in decreasing order. That is, the largest *G* − 1 weights are placed randomly in the secondary diagonal (elements *A*_*i*,*i*+1_), the next *G* − 2 largest weights are placed randomly in the ternary diagonal (elements *A*_*i*,*i*+2_), etc. (Note that the values *A*_*ij*_ in this case are only used to indicate that elements along diagonals are equal, but these values are not used to generate the weights.)

All four configurations are sparse, with only 2–4% of the putative edges being present in the graph. The *two negatively correlated blocks* structure has the same sparsity as the *complete* and *ar* graphs, but it has different weights of non-null mixture components. The *ar* graph has a more constrained structure, with weights among pairs of nodes which decay as |*i* − *j*| increases, for *i*, *j* ≤ *S*. Recall, however, that our method does not rely on any assumptions about the structure of the graph. Therefore, it is expected that its performance will only depend on the parameters involved in the mixture model. In the simulations presented here we examined the power of the method to detect edges in the graphs as a function of the location parameters of the log-normal components. We varied *θ*_1_, such that *θ*_1_ ∈ {−1.25, −1, −0.75, …, 0.75}, and set $\kappa_{1}^{2}=0.25$. In the *two negatively correlated blocks* configuration, we used *θ*_2_ = *θ*_1_ and *κ*_2_ = *κ*_1_. The weights which were generated according to the *L*_2_ *N* model were transformed into correlation coefficients using the tanh function, and the resulting covariance matrix was used to simulate 500 gene expression values for 100 subjects. For each configuration we generated 20 different datasets.

**Fig 2:**
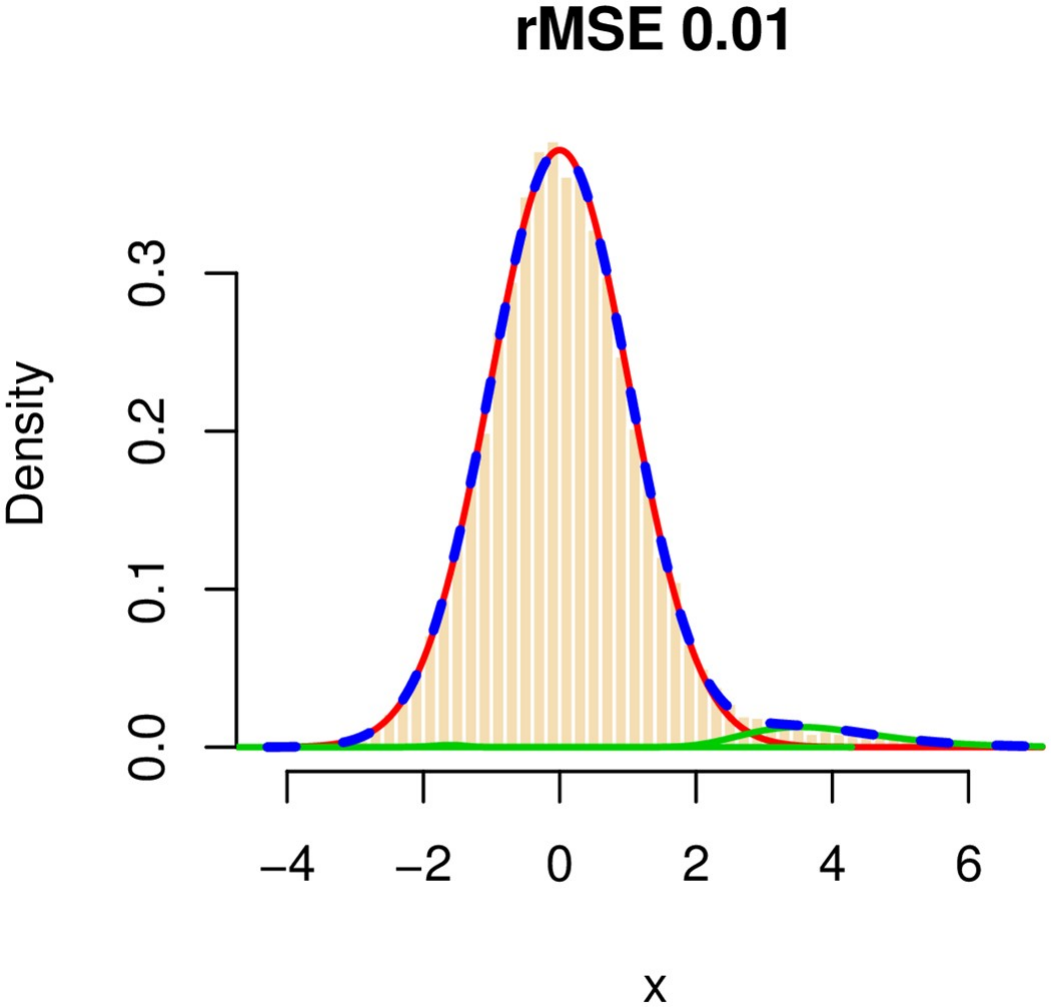
The distribution of *w*_*mn*_ = *arctanh*(*r*_*mn*_) for a simulated dataset. The number of genes is 500, of which 100 form a complete subgraph. The total number of edges in the graph is 4,950 (out of a total of 124,750 possible edges.) The red curve represents the null component, the green curves represent the non-null components, and the dashed blue line represents the fitted mixture distribution.

We applied our method and checked the goodness of fit of the mixture model and the ability to correctly recover the structure of the network, in terms of the number of true- and false-positive edges. In our simulations, we used the approach described in the statistical model and estimation section to control the false discovery rate at the 0.01 level. To demonstrate how well our algorithm estimates the true mixture model, we plotted for each configuration the histogram of the observed *w*_*mn*_ and the fitted mixture and measured the goodness of fit in terms of the root mean squared error (rMSE). In all configurations, the rMSE was very small (≤0.01), and the mixture weights (*p*_0_, *p*_1_, *p*_2_) were estimated very accurately and with increasing accuracy as *θ*_1_ increases. See, for example, S1 Fig in S1 File, where the average estimate of *p*_1_ is plotted versus *θ* using the *two negatively correlated blocks* configuration. A representative goodness of fit plot is shown in Fig 2, for the *complete* configuration, with *θ*_1_ = −0.25. The red curve represents the null component, the green lines represent the non-null components (in this case, *C*_2_ is estimated, correctly, to have a weight which is very close to 0), and the dashed blue line is the mixture.

**Fig 3:**
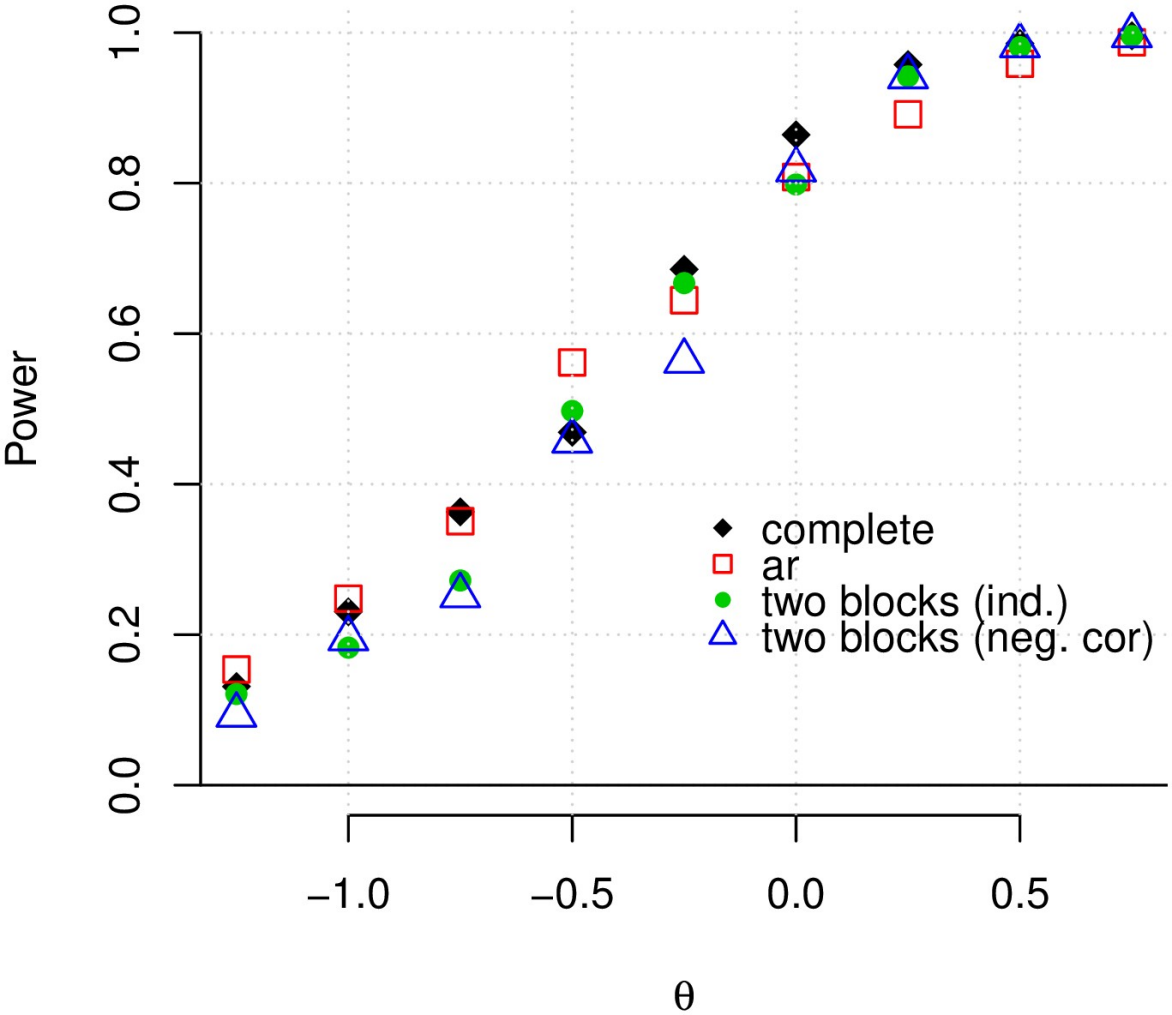
The average power of our method when the data are generated according to the *L*_2_ *N* mixture model. The average power is plotted against *θ* for a simulated dataset with 500 genes and four different forms of adjacency matrices (complete, autoregressive, two independent blocks, and two negatively correlated blocks). In all cases, the FDR was controlled at the 0.01 level.

Arguably, more important than assessing goodness of fit, is determining a method’s ability to recover the true network structure correctly, i.e. identify as many existing edges as possible while maintaining a low number of falsely-detected edges. Fig 3 shows the average power of our method to detect true edges for a range of values for *θ*_*i*_ (with a fixed *κ*^2^) and for different network configurations, where power is the total number of true-positives divided by the total number of edges in the graph. It can be seen that, as the location parameter of the log-normal distribution increases, the power increases and approaches 1. When the data are generated under the *L*_2_ *N* model, this is the expected behavior, since as *θ*_1_ (*θ*_2_) increases, the positive (negative) non-null component, *C*_1_ (*C*_2_), is pushed further to the right (left), making it easier to discriminate between non-null and null components. Note that our method has approximately the same power curve for all four configurations.

The average false discovery rate across all configurations and replications is 0.008. For small values of *θ*_1_ (< −0.75), the average FDR is slightly higher (approximately 0.012) and for *θ* > 0, the average FDR is less than 0.01. More detailed results regarding the achieved false discovery rate are shown graphically in S2 Fig in S1 File.

### Data generated under other models

In the second simulation study we evaluate the ability of our method to recover the true network structure and compare it with other methods. For a fair comparison, the data are not generated under *L*_2_ *N* model. Rather, we use data generated from a multivariate normal distribution whose covariance matrix Σ is defined as a function of an adjacency matrix *A*. In order to generate data, we use the R-package huge [39]. We generate five types of network configurations as follows:

- **random**: each edge is randomly set to exist in the graph using *K* i.i.d. Bernoulli(p) draws (*p* = 0.01, 0.05, 0.1). |*E*| ≈ 1000 × (1000 − 1) × *p*/2.
- **hub**: it consists of *g* disjoint groups, and nodes within each group are only connected through a central node in the group (*g* = 25, 50, 100). |*E*| = 1000 − *g*.
- **band**: an edge, *e*_*mn*_ between nodes *m* ≠ *n* is set to exist in the graph if 1 ≤ |*m* − *n*| ≤ *g* (*g* = 25, 50, 100). |*E*| = (2000 − 1 − *g*) × *g*/2.
- **scale-free**: scale-free networks are generated using the Barabási-Albert algorithm [40]. |*E*| = 1, 000.
- **overlapped-cluster**: we modified a function that generates a *cluster* network that consists of non-overlapping *g* groups. In the modified function, the groups are aligned in an adjacency matrix so that each group shares 20% of the nodes with its left-adjacent group and another 20% with its right-adjacent group. Edges in each group are randomly generated with probability *p*. We use *p* = 0.3 for *g* = 25 and 50, and *p* = 0.6 for *g* = 100. |*E*| ≈ (0.8 × (*g* − 1) + 1) × (1000/*g*) × (1000/*g* − 1) × *p*/2.

For each configuration, we generated expression profiles of *G* = 1, 000 genes for *N* = 70 samples. We compare our method with three existing methods: Meinshausen-Bühlmann graph estimation (**MB**, [24]), graphical lasso (**glasso**, [25]), and **correlation thresholding** graph estimation. For each of MB and glasso, we identify edges in two different ways. First, an edge *e*_*mn*_ between nodes *m* and *n* is estimated to exist (i.e. ${\hat{A}}_{mn}=1$) if the method chooses the node *m* as a neighbor of *n* **and** the node *n* as a neighbor of *m*, which we name as MB-AND and glasso-AND, respectively. Second, an edge *e*_*mn*_ is estimated to exist if the method chooses the node *m* as a neighbor of *n* **or** the node *n* as a neighbor of *m*, which we name as MB-OR and glasso-OR, respectively. The sparsity levels of MB and glasso are controlled by a regularization parameter λ and the correlation thresholding method by setting different thresholds from 0 to three times the true sparsity level.

We compare the performance in terms of the number of true positives (i.e. correctly identified edges) *given the **same** total number of edges identified*. We define the true network in two different ways: (i) based on the adjacency matrix—a pair *m*, *n* is set to be connected if and only if *A*_*mn*_ = 1, and (ii) by applying a threshold to the true covariance matrix—for a predetermined *t*, a pair is connected iff |Σ_*mn*_| > *t* where the threshold *t* is set to achieve the same sparsity level as that of the adjacency matrix. The two are different in that the former assumes conditional independence between two nodes that not connected by an edge, while the latter does not.

The results from the two network definitions are similar. We show the result from (i) whose true matrix is derived from the adjacency matrix, and show the result from (ii) whose true matrix is derived from the true covariance matrix in the (S3 Fig in S1 File).

**Fig 4:**
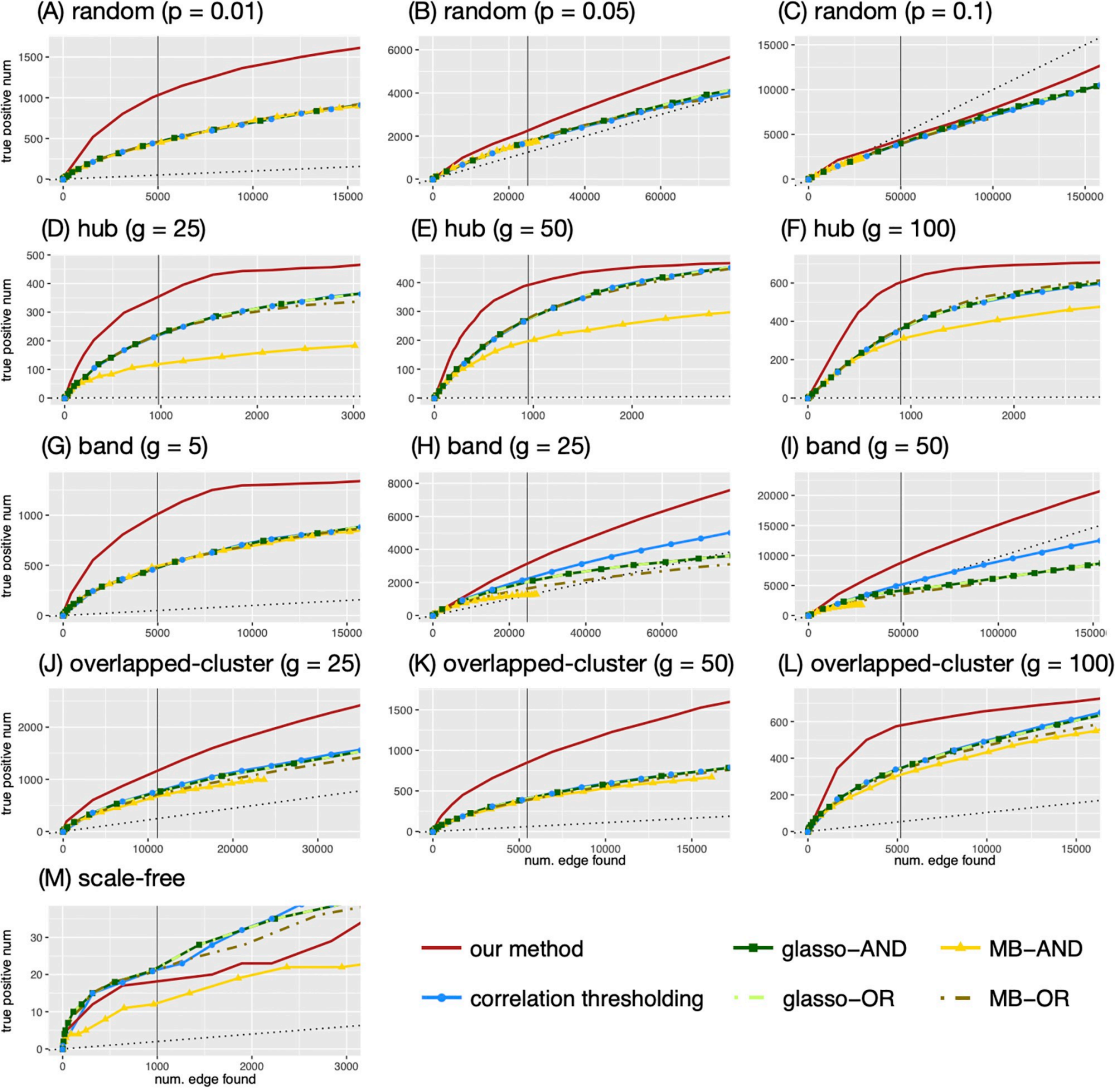
The numbers of true positive edges given the total number of edges identified by each method. The adjacency matrix used by the huge package is used to determine the true edges. The y-axis represents the number of true positive edges and the x-axis represents the total number of edges identified. The vertical line represents the number of true edges. The black dotted line is a regression line with 0 intercept and slope equal to the true sparsity, which represents the expected number of true positive edges when the edges are identified in a random manner (uniformly).

In Fig 4, we compare how our method performs in the different network configurations under the same network size (*G* = 1000). Each figure plots the number of true positive edges versus the total number of edges, colored by the different methods. Our method (solid red lines) outperforms all the other methods in the random, hub, band, and overlapped-cluster networks (see Fig 4A–4L). Note that for these configurations, the same qualitative results are obtained for other values of *G*. For example, in the hub, *g* = 100 configuration (panel F), the true number of edges is 900 (as indicated by the vertical line), and when our method detects 853 edges, 595 of them are true edges. In contrast, the thresholding method yields approximately 345 true edges out of the total 856 detected; MB-OR and glasso- yield approximately 370–380 true edges out of 900–960 edges detected; and MB-AND yields 312 true edges out of 930 edges detected. Notably, MB and glasso are comparable to, but in some cases worse than the correlation thresholding method in all network configurations.

The black dotted line in each plot represents the expected number of true positive edges when the edges are identified in a random manner. For example, in the band, *g* = 50 configuration (panel I), the competing methods do as well as or worse than chance, whereas our method gives much better results. In the random, *p* = 0.1 configuration (panel C), the other methods perform worse than choosing edges randomly and our method performs slightly better than chance to a certain point (approximately 30,000 total edges being detected) before deteriorating, but still gives better results than the other methods.

The scale-free configuration appears to be especially challenging for all the methods. They all detect only approximately 10–20 true edges when the total number of edges detected was 1,000 (Fig 4M). In this case, the correlation thresholding, glasso- and MB-OR are comparable to our method. While the other configurations show similar trends for different network sizes, the scale-free network shows different trends for different network sizes. In S4 Fig in S1 File, we depict the numbers of true positive edges given the total number of edges, obtained by each method in our comparison, for various network sizes of the scale-free network (*G* = 200, 500, 1000, 2000). We observe that the number of true positive edges tends to be smaller for all methods when *G* is larger. This is not unexpected, since the sparsity of a scale-free network increases with *G*, making the detection of a small number of true edges among a much larger number of putative ones, much more challenging. When *G* = 200, 500 (i.e., less sparse), our model still considerably outperforms the other methods. For example, when *G* = 200, our model identifies around 45 true edges out of 200 edges, while glasso-, MB-OR, and the correlation thresholding methods identify less than 30 true edges, and MB-AND identifies around 10 edges. For larger, and thus, more sparse networks, (e.g., *G* = 1000, 2000), the difference between the methods in terms of true positive edges is relatively small (about 2-4 edges).

## Case study—subtype characterization of breast cancer

We analyze a large dataset used by [41] who introduced SyNet, a computational tool aiming to improve network-based cancer outcome prediction. The dataset, referred to as ACES [42], contains expression data for 12,750 genes collected from 1,616 women across 12 studies. To obtain and preprocess the data, we use the raw data and scripts provided by [41] via their github page (github.com/UMCUGenetics/SyNet). We focus on analyzing gene networks by cancer subtype: Basal (n = 297), Her2 Positive (n = 191), Luminal A (n = 584), Luminal B (n = 440), and Normal (n = 104). Categorization into breast cancer types is based on the positive/negative status of three factors: estrogen receptor (ER), progesterone receptor (PR), and the number of copies of the HER2 gene. The Basal group is called the triple-negative breast cancer, and it includes tumor cells in which all three markers (ER, PR, HER2) are negative. The Her2 group includes cells which are ER and PR negative, but HER2 positive. The Luminal A group includes cells which are ER positive and PR positive, but HER2 negative. The Luminal B group includes cells which are ER positive, PR negative, and HER2 positive.

We use edgefinder to detect the edges in the network for each of the five groups. In order to assess the stability of the solution, in the sense of [24], and to avoid possible bias due to the different sample sizes, we fit the model 100 times using a different random sample of 80 subjects from each group in each iteration. The same edges are detected in each of the cancer groups in all 100 iterations, and in the normal group the same network is detected 99 times.

The mixture model for the normalized correlation coefficients fits all five subgroups very well, with rMSE = 0.01. For example, S5 Fig in S1 File shows the fitted curve for the Basal group.

With 12,750 genes there are 81,274,875 possible edges in the network. Controlling the false discovery rate at 0.001 edgefinder detects 28,628 edges in the network of the Normal group, 40,763 in the Basal group, 84,259 in the Her2 group, 52,919 in Luminal A, and 106,235 in Luminal B. Clearly, all five networks are very sparse (0.035-0.13% sparsity). However, in all five groups the edges are not at all random and they form several dense clusters. To define clusters we use two characteristics of nodes, namely the degree, *d*_*m*_, and the clustering coefficient, *γ*_*m*_, where *m* indicates a node *m*. Let *N*_*m*_ = {*n* | *A*_*mn*_ = 1} be the set of nodes adjacent to node *m*, and let *E*(*N*_*m*_) be the set of edges between nodes in *N*_*m*_. Then

$$\begin{array}{c} d_{m}=\left|N_{m}\right|\;\text{and}\;\gamma_{m}=\frac{\left|E(N_{m})\right|}{d_{m}\left(d_{m}-1\right)/2}\;. \end{array}$$

If *d*_*m*_ ≤ 1, the clustering coefficient is defined as *γ*_*m*_ = 0. By definition, *γ*_*m*_ ∈ [0, 1]. The degree of a node is interpreted as the involvement of the node in the network and the clustering coefficient as the connectivity among neighbors of the node. Note that *γ*_*m*_ *d*_*m*_ is, by definition, proportional to |*E*(*N*_*m*_)|/(*d*_*m*_ − 1), so it is interpreted as (approximately) the average degree among the neighbors of node *m*. Note also that *γ*_*m*_ *d*_*m*_ is bounded by *d*_*m*_.

We define highly-connected sub-graphs as ‘clusters’ using the following procedure. Initially, all nodes are classified as not being assigned to any cluster. Then, we find a node, *m*_1_, which yields the maximum sum *d*_*m*_ + *γ*_*m*_ *d*_*m*_ and set it as the central node in cluster 1. Cluster 1 consists of all the neighbors of *m*_1_. Then, we proceed iteratively by selecting a node which maximizes *d*_*m*_ + *γ*_*m*_ *d*_*m*_ among all nodes not already assigned to any cluster, and define a new cluster with this node (at the center) and all its neighbors. We can define a minimum cluster size to avoid working with too many small clusters, and in this application we set it to 30 nodes. We use the maximum value of *d*_*m*_+ *γ*_*m*_ *d*_*m*_ as the criterion to select central nodes because it gives preference to clusters which are both large and highly interconnected. Such clusters are more likely to be biologically meaningful than loosely connected clusters.

Using this approach we find 11, 13, 25, 28, and 15 clusters in the Normal, Her2, LumA, LumB, and Basal networks, respectively. We perform gene set enrichment analysis [30] and find the KEGG pathways significantly enriched in each cluster, in each subtype (we require FDR ≤ 0.01).

Fifteen pathways are enriched in *each* of the five subtypes in at least one cluster: ECM-receptor interaction, and Cell adhesion molecules from the ‘Environmental Information Processing’ category; Cell cycle, Oocyte meiosis, p53 signaling pathway, and Focal adhesion from the ‘Cellular Processes’ category; Hematopoietic cell lineage, Natural killer cell mediated cytotoxicity, T cell receptor signaling pathway, Chemokine signaling pathway, Progesterone-mediated oocyte maturation, and Protein digestion and absorption from the ‘Organismal Systems’ category; and Primary immunodeficiency, Staphylococcus aureus infection, Amoebiasis from the ‘Human Diseases’ category.

The other enriched pathways are summarized in Table 1. Thirteen pathways are enriched in each of the cancer groups, but *not* in the Normal group: Cytokine-cytokine receptor interaction (Environmental Information Processing), Phagosome (Cellular Processes), Antigen processing and presentation, and Intestinal immune network for IgA production (Organismal Systems), and the following nine from the ‘Human Diseases’ category: Rheumatoid arthritis, Autoimmune thyroid disease, Allograft rejection, Graft-versus-host disease, Viral myocarditis, Type I diabetes mellitus, Hepatitis C, Toxoplasmosis, and Leishmaniasis. It is interesting that a large number of human disease pathways form highly-connected clusters in each of the cancer subtypes but not in the normal cohort. In particular, two of the pathways correspond to autoimmune diseases and two other correspond to graft rejection. It is plausible that the body’s response to breast cancer is similar to its response to these, and perhaps related diseases, in terms of changing the production rate of specific proteins. Such insights have the potential to yield targeted therapies, but this is beyond the scope of this paper.

**Table 1:** Gene set enrichment analysis.

	Normal	Her2	LumA	LumB	Basal
**Metabolism**					
00190 Oxidative phosphorylation			X		
00140 Steroid hormone biosynthesis		X			
00380 Tryptophan metabolism		X			
00982 Drug metabolism—cytochrome P450		X			
**Genetic Information Processing**					
03040 Spliceosome			X		
03030 DNA replication		X		X	
**Environmental Information Processing**					
04630 Jak-STAT signaling pathway		X			
04080 Neuroactive ligand-receptor interaction			X		X
04060 Cytokine-cytokine receptor interaction		X	X	X	X
**Cellular Processes**					
04145 Phagosome		X	X	X	X
04140 Autophagy—animal			X		
04530 Tight junction			X		
**Organismal Systems**					
04610 Complement and coagulation cascades		X	X	X	
04620 Toll-like receptor signaling pathway			X	X	
04622 RIG-I-like receptor signaling pathway		X		X	
04612 Antigen processing and presentation		X	X	X	X
04662 B cell receptor signaling pathway	X	X		X	X
04664 Fc epsilon RI signaling pathway			X	X	X
04666 Fc gamma R-mediated phagocytosis			X	X	
04670 Leukocyte transendothelial migration			X	X	
04672 Intestinal immune network for IgA production		X	X	X	X
03320 PPAR signaling pathway	X		X		
04740 Olfactory transduction	X	X	X		X
04380 Osteoclast differentiation	X	X	X	X	
**Human Diseases**					
05310 Asthma			X	X	X
05322 Systemic lupus erythematosus			X	X	
05323 Rheumatoid arthritis		X	X	X	X
05320 Autoimmune thyroid disease		X	X	X	X
05330 Allograft rejection		X	X	X	X
05332 Graft-versus-host disease		X	X	X	X
05010 Alzheimer disease			X		
05012 Parkinson disease			X		
05016 Huntington disease			X		
05410 Hypertrophic cardiomyopathy (HCM)			X		
05414 Dilated cardiomyopathy (DCM)			X		
05416 Viral myocarditis		X	X	X	X
04940 Type I diabetes mellitus		X	X	X	X
05160 Hepatitis C		X	X	X	X
05145 Toxoplasmosis		X	X	X	X
05140 Leishmaniasis		X	X	X	X
05142 Chagas disease (American trypanosomiasis)			X		

**Fig 5:**
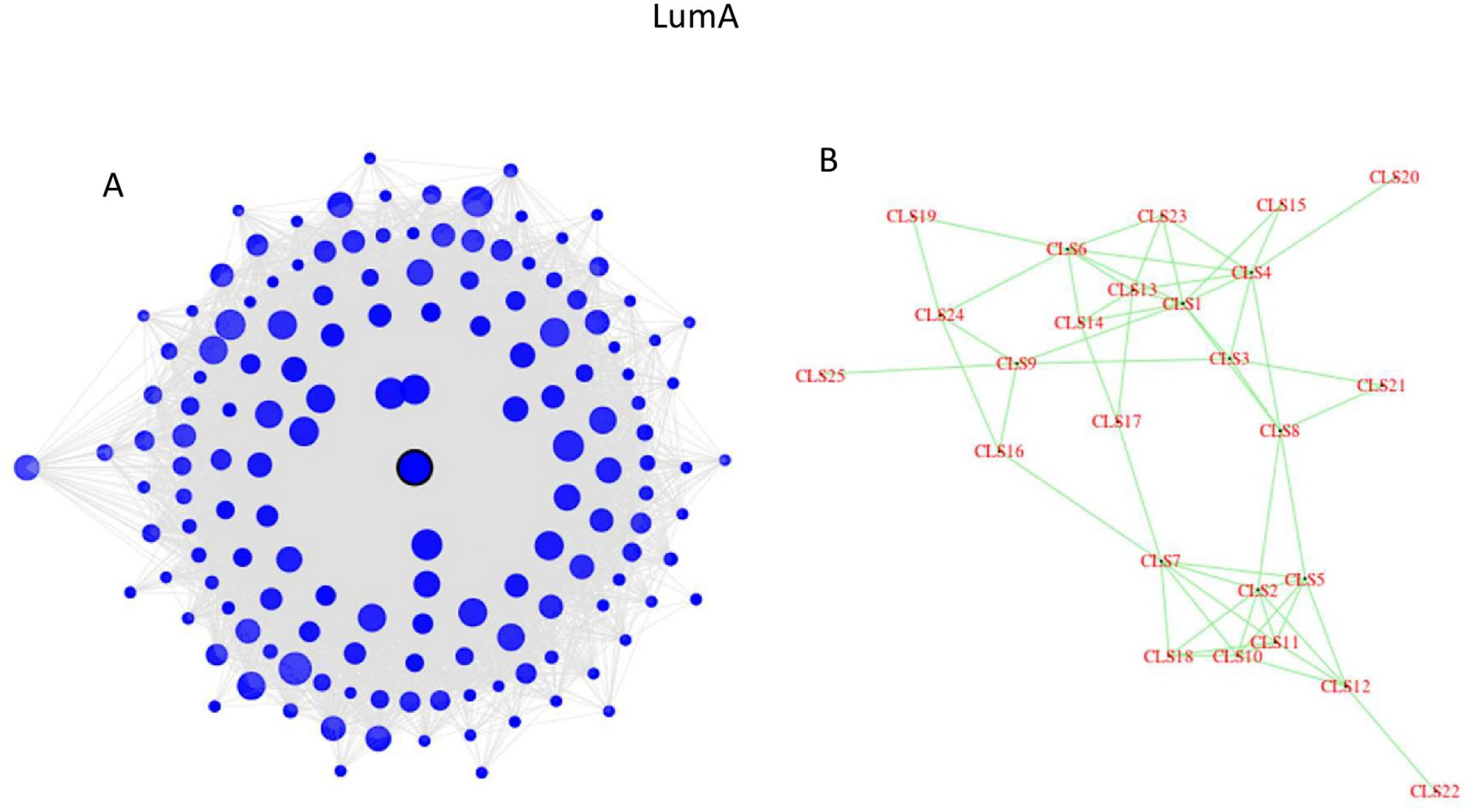
The LumA network analysis: (a) The structure of cluster #3 which is enriched in Alzheimer, Parkinson, and Huntington diseases, Hypertrophic cardiomyopathy (HCM) and Dilated cardiomyopathy (DCM), and Chagas disease. (b) Each cluster consisting of 30 or more genes is depicted as a single point.

Ten pathways are uniquely enriched in the **LumA subtype**, of which six are in the ‘Human Diseases’ category: Alzheimer, Parkinson, and Huntington diseases, Hypertrophic cardiomyopathy (HCM) and Dilated cardiomyopathy (DCM), and Chagas disease. Fig 5 depicts some characteristics of the LumA network, as detected by edgefinder. Panel B (right) shows the connections among the 25 clusters in the LumA network and we see that they form a connected graph. Among the 25 clusters in the LumA network only one is enriched in the human diseases category. This cluster contains 149 genes and is highly interconnected, as can be seen in Fig 5A. The central node in the cluster is CD53 (Entrez 963, Leukocyte surface antigen CD53 protein), a suppressor of inflammatory cytokine production which was shown to be a regulator of immune cell function [43–45]. The radius of each circle in Fig 5A represents the degree of the node, and the distance from the center corresponds to the relative dissimilarity with the central node, measured in terms of which neighbors the gene and the central node do *not* have in common. The shade of the circles represents the percentage of neighbors of a node which are in the same cluster. In this case, the dark shade of most of the points indicates that the nodes in this cluster are mostly connected to other nodes in the same cluster. The median degree (number of neighbors) in this cluster is 75, and three quarteres of the nodes have at least 47 neighbors. Furthermore, for 75% of the genes in the cluster, at least three quarteres of their neighbors in the same cluster, and 25% of the genes in this cluster have at least 90% of their neighbors in the same cluster.

**Fig 6:**
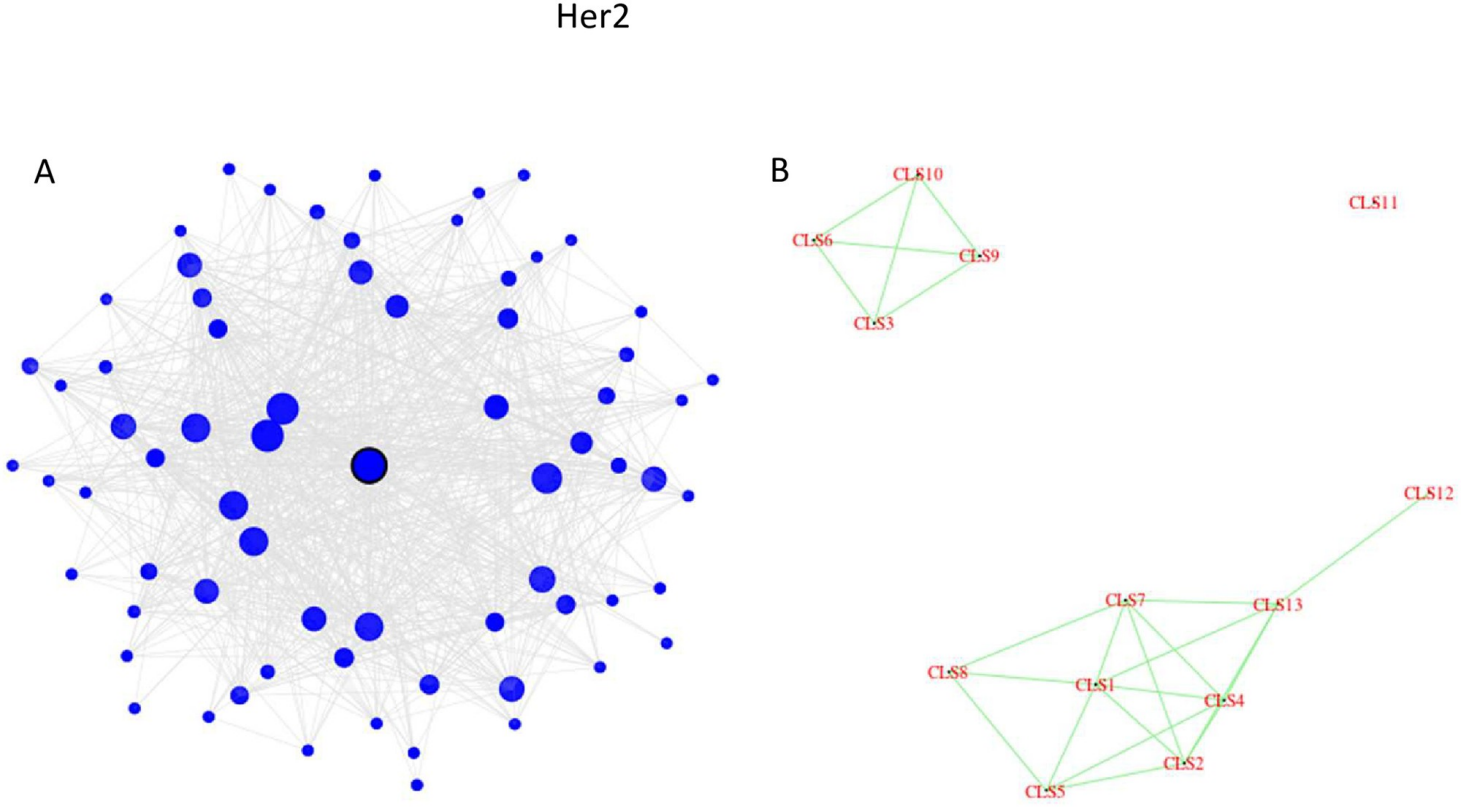
The Her2 network analysis. (a) The structure of cluster #11 which is enriched in three metabolic pathways. (b) Each cluster consisting of 30 or more genes is depicted as a single point.

Four pathways are uniquely enriched in the **Her2 subtype**, three of which are in the Metabolism category (Steroid hormone biosynthesis, Tryptophan metabolism, and Drug metabolism—cytochrome P450). These three metabolic pathways all belong to a single cluster in the Her2 network. This cluster is depicted in Fig 6A, and the entire network is depicted in Fig 6B, where each cluster (consisting of at least 30 genes) is represented by a single point. This ‘drug metabolism’ cluster contains 70 genes, with SLC26A3 at the center, which has been identified as a marker of resistance to neoadjuvant chemotherapy in HER2-negative breast cancer [46]. The cluster is very interconnected, with a minimum of 58% within-cluster connections. For three quarters of the genes in this cluster, more than 80% of their neighbors are in the same cluster. Graphically, it is demonstrated by the dark shade of the nodes in Fig 6A. Furthermore, Fig 6B shows that the Her2 graph consists of three disconnected components, and this ‘drug metabolism’ cluster (#11 in the plot) is isolated from all other clusters in the Her2 network. The relationship of *drug metabolism–cytochrome P450* to HER2 has been reported in [47], the relationship of *steroid hormone biosynthesis* to HER2 has been reported in [48], and the relationship of *tryptophan metabolism* to HER2 has been reported in [49, 50].

## Discussion

We propose a new approach for detecting edges in gene networks, based on co-expression data. We consider the entire set of genes as a network in which nodes represent genes and weights on edges represent the correlation between expression levels of pairs of genes. We start by modeling the normalized pairwise correlations as a mixture of three components: a normal component with mean 0, representing the majority of pairs which are not co-expressed, and two non-null components, modeled as log-normal distributions, for positively and negatively correlated pairs.

From a theoretical point of view, the so-called *L*_2_ *N* model has the advantage that the overlap between the null component and the non-null components around 0 is negligible. This helps to avoid identifiability problems which are known to affect other mixture models which rely only on normal components, such as the spike-and-slab or a three-way normal mixture model. Furthermore, the mixture model allows us to accurately estimate the proportion of spurious correlations among all pairs of genes and to derive a cutoff criterion in order to eliminate the vast majority of the edges in the graph that correspond to uncorrelated genes. We also derived estimators for the probabilities of Type-I and Type-II errors, as well as the false discovery rate associated with the cutoff criterion.

From a practical point of view, this model appears to fit co-expression data extremely well, even when the data are not generated according to the mixture model. Our simulation study systemically evaluated the power, false discovery rate, and goodness of fit of our model, as implemented in the edgefinder package, and compared its ability to discover the network structure with other methods. Using various well known biological network configurations, the simulations demonstrated that edgefinder outperformed the other methods in most of the network configurations, illustrating its potential as a valuable tool for network structure estimation. Estimation of the model parameters is done very efficiently, using the EM algorithm. In typical gene expression datasets which consist of thousands of genes and millions of putative edges, computational efficiency is critical.

Our approach does not require any assumptions about the underlying structure of the network. We only assume that the normalized correlations follow the *L*_2_ *N* model. This is a very modest assumption since the Fisher z-transformed correlations are indeed (asymptotically) normally distributed for all the uncorrelated pairs.

Our case study yielded results that are insightful and consistent with previous findings. We find thirteen pathways which are enriched in each of the cancer groups but not in the Normal group, with two of the pathways associated with autoimmune diseases and two other with graft rejection. We also find specific characteristics of different breast cancer subtypes. For example, the Luminal A network includes a single, highly connected cluster of genes, which is enriched in the human diseases category. The central node in the cluster is CD53, a suppressor of inflammatory cytokine production which was shown to be a regulator of immune cell function. In the Her2 subtype network we find a distinct, and highly interconnected cluster which is uniquely enriched in drug metabolism pathways. At the center of this cluster we found a gene which has been found to be a marker of resistance to neoadjuvant chemotherapy in HER2-negative breast cancer (SLC26A3). Causality cannot be determined from the data we have used, but our results may help to perform subsequent analyses in order to explore the connection between cancer and a weaken immune system, and perhaps lead to personalized treatments.

In summary, our novel approach provides a powerful way to estimate sparse gene networks from co-expression data. Our approach provides several theoretical and practical benefits. The *L*_2_ *N* mixture model that our method uses for modeling the gene networks, borrows strength across pairs of genes, resulting in a better power in detecting the edges. Our approach can be universally used to any network configurations, as it only uses a very modest assumption about gene correlations. We also provide a computationally efficient estimation approach using EM algorithm, and allows users to control the error rate.

We plan to extend this method to handle time varying networks. This will be particularly useful when analyzing gene expression data from repeated measures designs. This is very important in longitudinal studies, where the question of interest may be how gene networks change over time, and whether such changes are determined by other factors, such as treatment, age, etc. For a survey of link prediction methods, especially in the context of network evolution, see [51]. This is not trivial because, repeated measurements obtained from the same subject are likely to exhibit a high degree of correlation within subject across most, or all genes. Thus, the model has to be extended in order to account for such experimental designs.

We also plan to extend the model to applications that involve multiple platforms, such as methylation and proteomics. In principle, with the appropriate normalization technique for each platform, one can simply construct a graph with *G*_1_ + … + *G*_*k*_ nodes, where *G*_*i*_ is the number of ‘building blocks’ observed in each platform. However, much more work is needed to establish the theoretical framework to define ‘co-expression’ across platforms. This type of extension will also require developing a method to detect subtle network changes. One possible direction is to investigate whether the approach of [52] can be integrated with ours. This may not be straightforward since our method relies on sparsity in the correlation matrix and an empirical Bayesian mixture model while [52] rely on the difference of two precision matrices and finding edges with the lasso and the so-called D-trace loss function.

## Supporting information

S1 File (PDF)
