# Supplementary material for: A mixture model to detect edges in sparse co-expression graphs with an application for comparing breast cancer subtypes
Source: PLoS One. 2021 Feb 11;16(2):e0246945. doi: 10.1371/journal.pone.0246945 (PMC7877669; doi:10.1371/journal.pone.0246945)
Supplement: S1 File — (PDF) [file pone.0246945.s001.pdf]

S1 File

Supplementary Materials for 'A mixture model to detect edges in sparse co-expression graphs with an application for comparing breast cancer subtypes' by Haim Bar and Seojin Bang.

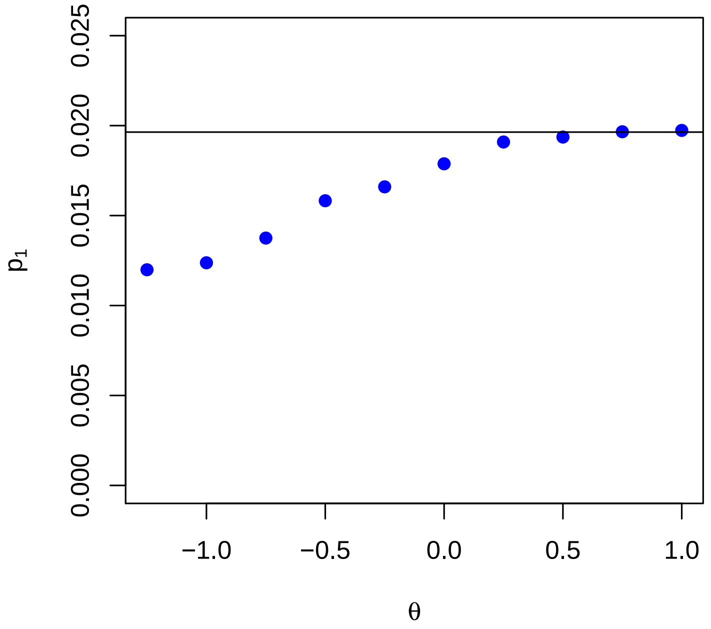

S1 Fig: The average estimate of p_1, the proportion of positively correlated pairs of genes, from 20 replications, is plotted versus θ in the *two negatively correlated blocks* configuration. The horizontal black line depicts the true value of p_1.

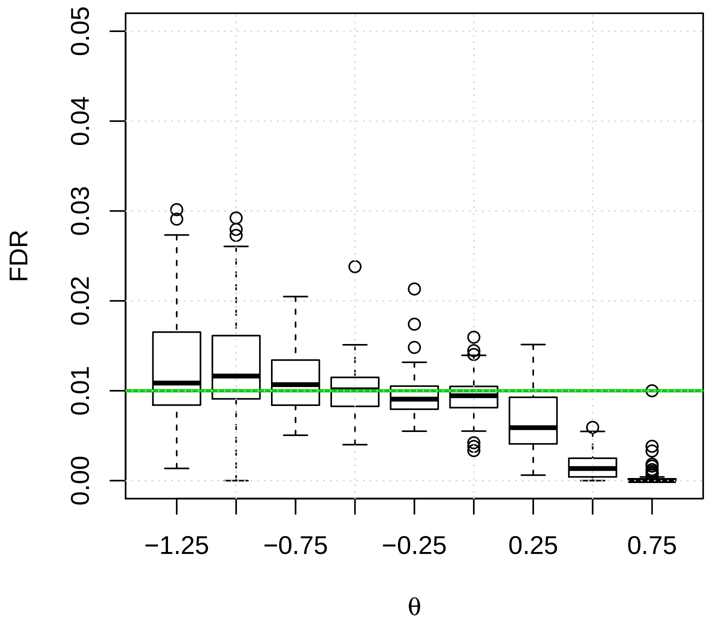

S2 Fig: The observed false discovery rate in the simulations under the L_2N model, as a function of the location parameter of the log-normal distributions of the non-null components. The green horizontal line shows the level (0.01) which we used to control the false discovery rate. Using the notation from the paper we determined the thresholds, c_1 and c_2, that correspond to this FDR level. The boxplots show the distributions across four network structures, with 20 replications in each configuration.

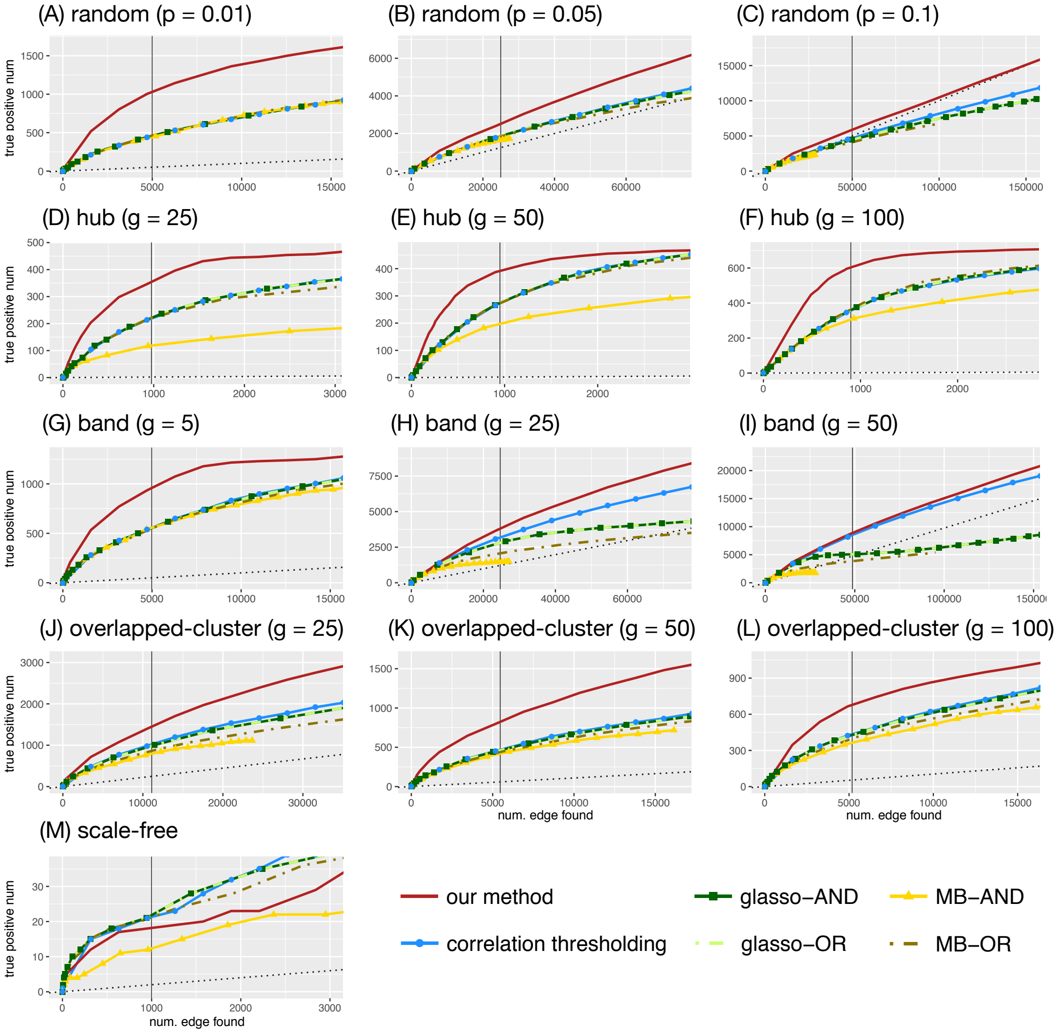

S3 Fig: The numbers of true positive edges given the total number of edges identified by each method. The true matrix is obtained by applying a threshold to the true covariance matrix. The y-axis represents the number of true positive edges and the x-axis represents the total number of edges identified. The vertical line represents the number of true edges. The black dotted line is a regression line with 0 intercept and slope equal to the true sparsity, which represents the expected number of true positive edges when the edges are identified in a random manner.

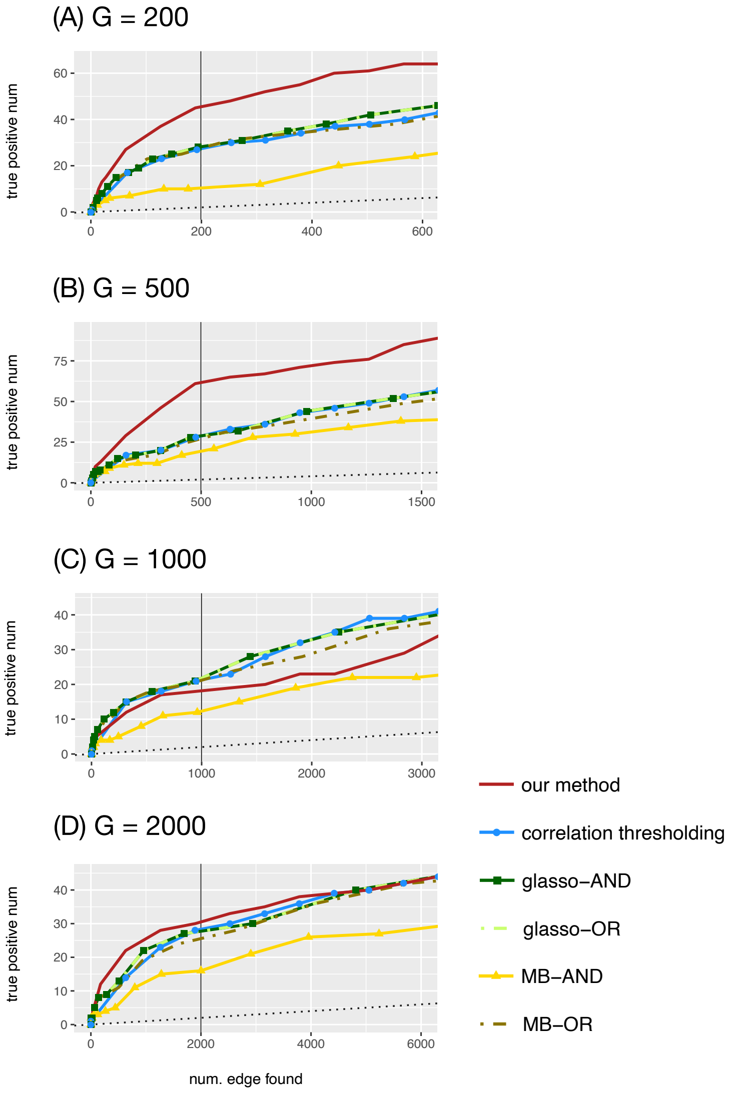

S4 Fig: The numbers of true positive edges given the total number of edges using three scale-free network configurations ($G = 200, 500, 1000, 2000$, where G is the number of genes). The adjacency matrix is used as the true matrix. The y-axis represents the number of true positive edges and the x-axis represents the total number of edges identified. The vertical line represents the number of true edges. The black dotted line is a regression line with 0 intercept and slope equal to the true sparsity, which represents the expected number of true positive edges when the edges are identified in a random manner.

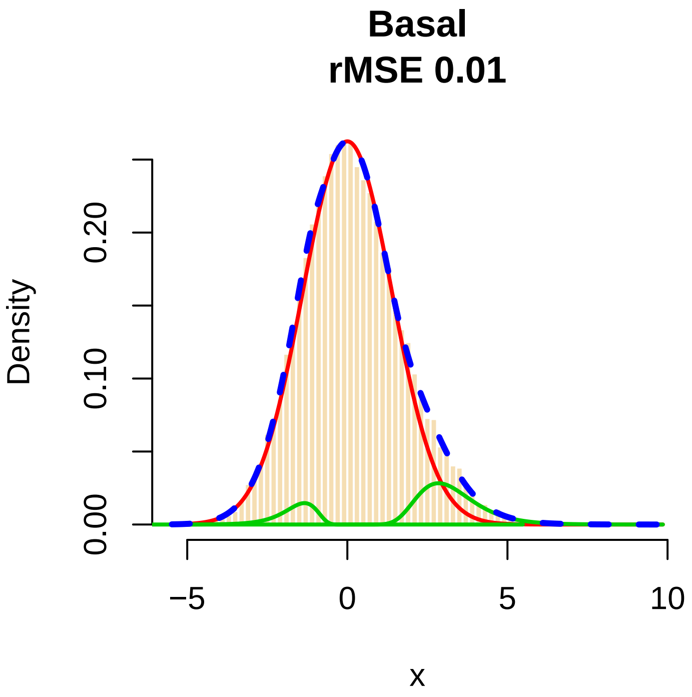

S5 Fig: Goodness of fit plot of L_2N mixture model. The distributions of $w_{mn} = arctanh(r_{mn})$ for the Basal group. The red curve represents the null component, the green curves represent the nonull components, and the dashed blue line represents the fitted mixture distribution.
